# Interactive Manipulation of Nonconductive Microparticles in Scanning Electron Microscope by a Virtual Nano-Hand Strategy

**DOI:** 10.3390/mi10100670

**Published:** 2019-10-02

**Authors:** Mei Liu, Kai Cheng, Xiangzheng Qin, Zhenzhong Wei, Brandon Borom, Weilin Su, Jinbo Chen, Yunpeng Feng, Tao Wang, Jinjun Rao

**Affiliations:** 1 Shanghai Key Laboratory of Intelligent Manufacturing and Robotics, School of Mechatronic Engineering and Automation, Shanghai University, Shanghai 200072, China; mliu@shu.edu.cn (M.L.); Caine_Cheng@shu.edu.cn (K.C.); wdmzjqxz@163.com (X.Q.); wwzhuiwan@shu.edu.cn (Z.W.); WEILINSU3@163.com (W.S.); flashpoint156@126.com (J.C.); 2 Department of Nutrition Science and Dietetics in the College of Agriculture, Biotechnology, and Natural Resources, University of Nevada, Reno, NV 89557, USA; cloverbmassage@gmail.com; 3 Shenzhen Research Institute, Beijing Institute of Technology, Shenzhen 518000, China; roc@bit.edu.cn

**Keywords:** scanning electron microscope (SEM), interactive micromanipulation, nonconductive microparticle, virtual nano-hand strategy

## Abstract

Micro/nano-manipulation is the fabrication of particular constructs on devices at the micro/nano-scale. Precise manipulation of microparticles is one of the key technological difficulties in manufacturing micro/nano-scale components. Based on scanning electron microscopy and nanomanipulator, this paper adopts a direct push method to operate randomly distributed microparticles into ordered structures. A two-probe interaction strategy is proposed to enable microparticle movements in all directions efficiently and avoid scratching the substrate surface. To overcome the uncertainties in micromanipulation, a virtual nano-hand strategy was also implemented: long-range advance of each microparticle is realized by multiple single-step pushes, whose trajectory is theoretically analyzed. The pushes are well programmed to imitate effects of a more powerful and determined hand. Experimental results show that the theoretical single-step motion trajectory is in line with actual operation, and the proposed strategy can ensure precise operation of the microparticles in all directions and improve reliability and effectiveness of operation.

## 1. Introduction

Micro/nano-operation refers to the measurement of physical, chemical and biological properties of objects at micro/nano-scales, and manufacturing micro/nano-systems/devices with new functions by pushing-pulling, extracting, handling and placing of micro/nanoobjects, such as micro/nanoparticles and nanowires. The new micro/nano-systems/devices provide new opportunities and technological approaches for developments of electronics, information science, materials, advanced manufacturing and biomedical fields [1,2].

At present, atomic force microscopes (AFM) and scanning electron microscopes (SEM) have become the most popular equipment for micro/nano-operations. AFM has high resolution and motion precision, controllable tools mode, good repeatability and various applicable environments. AFM has been able to perform precise operations and the assembly of carbon nanotubes, single-molecule viruses, etc. [3,4,5,6,7,8]. However, AFM cannot image and operate simultaneously, as its control strategy of acting after observation and limited scanning range both limit the operating efficiency, as scanning of each frame takes a relative long time [9]. SEM has a large, built-in vacuum operating environment and real-time imaging capability, and can be equipped with a nanomanipulator, which works with piezoelectric drive and multi-degree freedom. It can carry on mechanical and electrical property measurement on nanomaterials, nano-electromechanical system (NEMS), assembly, cell characterization and manipulation [9,10,11,12,13,14,15,16,17]. SEM can also be combined with AFM, focused ion beam (FIB), etc. to achieve a more powerful ‘nano-laboratory’ for imaging, automated operation and characterization, simultaneously [18,19,20].

In order to improve operation reliability and efficiency, researchers have proposed many algorithms and techniques, such as augmented reality in AFM [21], local scanning [22,23], feature-based tip localization and planning in nanomanipulations [24], random positioning [24,25] tc. Various nanomanipulation protocols have also been proposed, which are mainly categorized into two types: push and pick-and-place [9,26]. As for pushing with AFM tips, Kim et al. developed a tapping protocol, where a nanoparticle is kicked to reduce the static friction, and then the nanoparticle is dribbled to a target position in tapping mode [27]. Zhao et al. implemented a successive directional push protocol [28]. Xu et al. proposed a sequential parallel pushing (SPP) algorithm for AFM [29]. Those methods were designed to work without precise information about the particle position. Although these methods have improved the operation efficiency, particle loss occurs inevitably during operation. J. Hou et al. proposed a ‘virtual nano-hand’ protocol, where a single-tip mimics the manipulation effect that multi-AFM tips can achieve, by planned high speed sequential tip pushing [7,30]. The manipulation yield was much improved.

The pick-and-place manner is another manipulation technique, and is good for long-distance movement, whose operation scale is only limited by the manipulator travel limit [31,32]. Decossas et al. successfully used AFM tips to pick up silicon nanocrystals from a Si surface and move to the target location and release them by a series of pulses [33]. Cao et al. used the pick-and-place strategy to manipulate microparticles by one nanomanipulator tip inside SEM [34]. E-beam irradiation was utilized to glue and pick up microparticles, and releasing was accomplished by adjusting the end tilt angle. However, the success rate of the pick-and-place protocol still needs to be improved, as the repeatability of E-beam irradiation pick-up is not very reliable. 

In this paper, a direct push protocol based on SEM and nanomanipulator was implemented, which is tunable in real-time, and able to transport microparticles to a target position reliably. In order to enable precise movement of microparticles in all directions, and avoid substrate scratching, a more efficient double-tip operation skill was proposed. In addition, the virtual nano-hand strategy was adopted to improve reliability: the long-distance movement was decomposed to multiple short pushes, and a single-tip was planned to mimic the manipulation effect of a ‘hand’ by designed sequential pushing. During the motion, operation parameters, such as the interaction position was adjusted in time, and the motion error was kept in an allowed limit. We demonstrated, by experiments, that this new method is efficient and convergent. Two example patterns were fabricated using our system, and the entire operation process can be observed in real-time.

## 2. Theoretical Modeling Analysis

**Figure 1:**
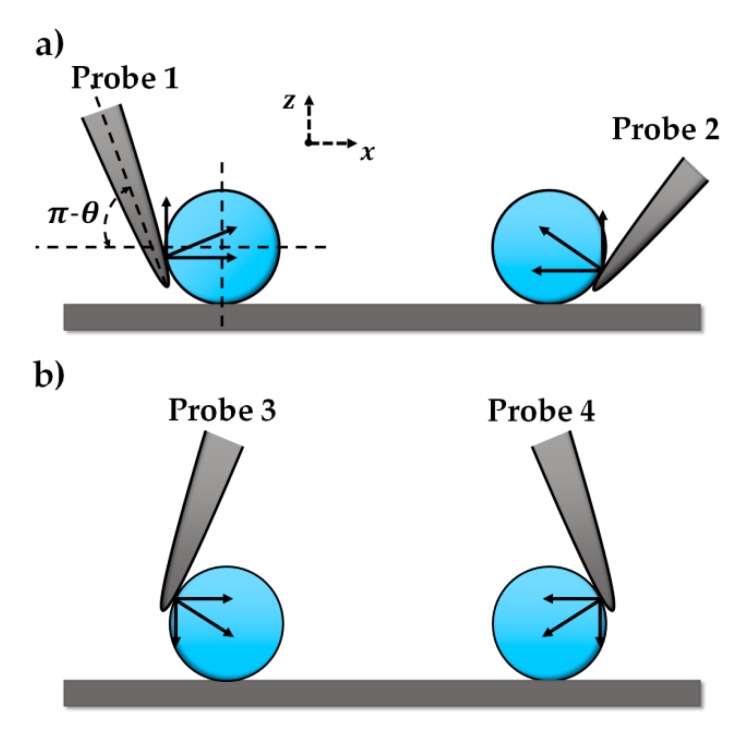
Interactions between the probe tip and microparticles. *θ* is the angle between the tip centerline and *x*-axis. (**a**) The tips move microparticles in a push mode, where an upward force is applied on the microparticles. (**b**) The tip moves microparticles in a drag mode, where a downward force is applied on the microparticles.

During the tip–microparticle interaction, poses of the tips are fixed. Push angle *θ*, i.e., the angle between the probe center line and *x*-axis, is also set. As shown in Figure 1, probe 1 could push the microparticle right, while probe 2 could push the microparticle left. Nevertheless, for cases in Figure 1b, where the tips move microparticles in a ‘drag’ mode, the interaction will inevitably generate a normal pressure on the microparticle and the substrate, probably causing stress or even damage to the substrate. Our double-tip operation skill is that probe 1 pushes the microparticle to the right, and *θ* > 90°; the opposite probe 2 pushes the microparticle to the left, and *θ* < 90°, as shown in Figure 1a. The two-probe operation not only protects the substrate from damage by avoiding pressure on the substrate, but also increases operation efficiency by reducing tip paths compared to a single-tip operation.

**Figure 2:**
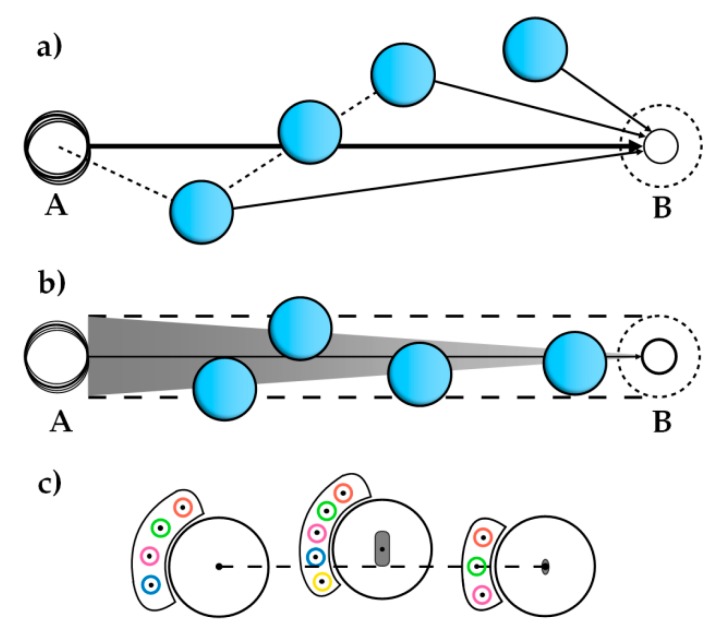
Different manipulation protocols. (**a**) The manipulation process of target-oriented push (TOP), where the microparticle is always pushed toward the target. (**b**) Virtual nano-hand strategy, where the push direction is always parallel to $\vec{AB}$. (**c**) Stable pushing of the microparticle with the nano-hand effect.

In a SEM vacuum chamber, due to many uncertainties, such as creep, hysteresis, temperature drift, etc., it is difficult to guarantee that the microparticle center locates on the pushing path. The tip can only apply a point force to the microparticle, as its touch area is small. Therefore, microparticle losses often occur, that is, the probe slides over the microparticle and the two separate. To this end, this paper adopted a virtual nano-hand strategy to improve reliability and repeatability, as shown in Figure 2.

### 2.1. Theoretical Single-Step Trajectory of a Microparticle 

**Figure 3:**
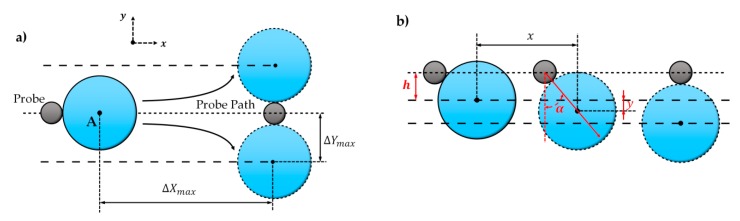
Single-step microparticle trajectories with different contact positions. (**a**) The microparticle center locates on the tip push path. (**b**) The microparticle center deviates from the tip push path. α is the angle between the vertical direction and tip–microparticle link line, denoting the transient moving direction of the microparticle. *h* is the initial vertical distance between the microparticle and tip push path. Point Zero locates at the initial position of the microparticle.

The single-step motion of the microparticle is defined as its movement between the initial contact and separation, as shown in Figure 3. If the interaction position of the tip–microparticle is different, the microparticle motion trajectory will also be different.

If the microparticle center locates on the push path $\vec{AB}$ (the probability is very low), the tip and microparticle will show a maximum displacement $\Delta x_{max}$ and $\Delta y_{max}$ ($\Delta y_{max}=R_{p}+r_{p}$, *R*_p_ and *r*_p_ are radius of the microparticle and tip horizontal section at the contact point), as shown in Figure 3a. If the microparticle center deviates from the push path $\vec{AB}$ by h (this probability is high), as shown in Figure 3b, the one-step displacement in the *x* and *y* directions will become Δ*x* and Δ*y* $(\Delta y=\Delta Y_{max}-h$).

Ignoring rotation movements, the transient push-in angle *α* (the angle between the *y*-axis and the microparticle–tip center line, as shown in Figure 3b, in the single-step motion is:

(1) $$\alpha =\arccos\left(\frac{h+y}{\Delta Y_{max}}\right)$$

where *y* is present displacement of the microparticles in vertical direction. The whole single-step motion could be divided into a combination of numerous micromotions. In each micromotion stage, the microparticle moves along with the angle *α*, thus the micromotion *dx* and *dy* in *x* and *y* will be related as [35]:

(2) dx=tanα·dy

Therefore, the microparticle motion trajectory can be integrated as:

(3) $$x=\int_{0}^{y}\frac{\sqrt{\Delta Y_{max}{}^{2}-{\left(y+h\right)}^{2}}}{y+h}dy$$

where *x* is present displacement of the microparticles in horizontal direction, and can be solved analytically as:

(4) $$\begin{array}{l} x=\sqrt{\Delta Y_{max}{}^{2}-{\left(y+h\right)}^{2}}-\sqrt{\Delta Y_{max}{}^{2}-h^{2}}\\ \;-\Delta Y_{max}\left[\mathrm{atanh}\frac{\sqrt{\Delta Y_{max}{}^{2}-{\left(y+h\right)}^{2}}}{\Delta Y_{max}}-\mathrm{atanh}\frac{\sqrt{\Delta Y_{max}{}^{2}-h^{2}}}{\Delta Y_{max}}\right] \end{array}$$

With this equation, the microparticle motion trajectory (y∈(0~Δy)), with different offset h, can be calculated.

### 2.2. Virtual Nano-Hand Strategy 

This paper adopts the virtual nano-hand strategy initially developed on AFM. The working principle has been introduced in previous publications [6,27]. Specifically, the entire movement process is divided into a series of single pushes. The trajectories of each push can be predicted with Equation (4). The current interaction position is chosen based on the relative position between the microparticle and the theoretical push path $\vec{AB}$. The push direction is always parallel to $\vec{AB}$.

As shown in Figure 2, different from the target-oriented push (TOP) strategy, the virtual nano-hand approach is: if the microparticle center is over $\vec{AB}$, the next interactive position will be selected over $\vec{AB}$; if the microparticle is below $\vec{AB}$, the next interactive position will be set below $\vec{AB}$. The previous error could be amended by next movements. By contacting multiple contact points, a virtual nano-hand is formed, which kept the microparticle within allowed error. Correspondingly long-range microparticle movement would be realized under uncertain conditions. In the whole intermediate process, no precision location information is needed, and the operation time is greatly reduced. 

## 3. Experiments

**Figure 4:**
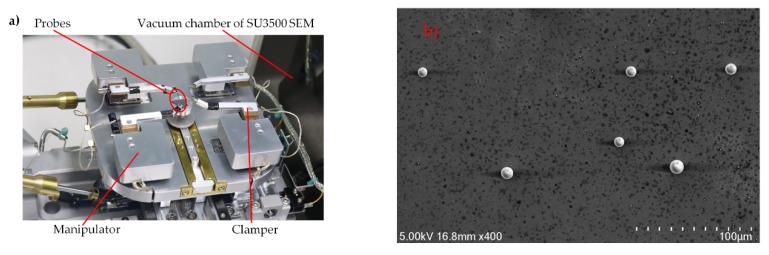
(**a**) Experimental setup, consisting of a nanomanipulator clamping two probes, embedded into scanning electron microscope (SEM) vacuum chamber. (**b**) A random distribution of microparticles on the substrate.

The experimental setup is shown in Figure 4a. The nanomanipulator (Toronto Nano Instrumentation Inc, NB202-SEM, Toronto, Canada) was installed in the vacuum chamber of SEM (SU3500, Hitachi, Japan). The manipulator had a coarse and fine three-degree-of-freedom (DOF) mode that could be switched in real time according to operational tasks. The coarse positioner (SL0610, SmarAct, Oldenburg, Germany) had a 10 mm stroke and a resolution of 100 nm; the fine piezoelectric grade positioner (AE0203D04F, Thorlabs, Newton, NI, USA) had a stroke of 20 mm and a resolution of 1nm. The probe model was ST-20-0.5 from GGB Industries Inc, with a tip diameter of 0.5 μm and taper of 10°. Glass microparticles with a diameter of 10 ± 2 μm were used as experimental samples.

Several tests of manipulating microparticles were carried out to verify the effectiveness of the double-tip and virtual nano-hand strategy, also shown in supplementary videos (Videos S1 and S2). First, the microparticles were diluted with pure ethanol solution, and 1 mL of the solution was pipetted onto the silicon substrate and dried for one hour in a clean chamber. The substrate was then placed on the nanomanipulator stage. Two probes were mounted on the nanomanipulator before placed in SEM. Tip pushing speeds were set as 2 μm/s, and the contact angle, θ, was set as 5π/6 (as shown in Figure 1). Since SEM lacks depth information in the *Z* direction, to avoid possible collision between the nanomanipulator tip and the substrate, their image sharpness and relative motion were combined for effective contact detection.

**Figure 5:**
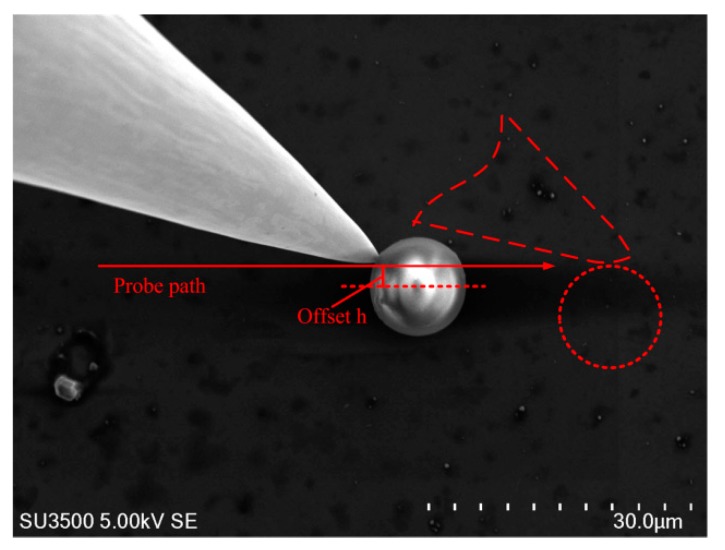
Tip–microparticle interaction. Tip push path and offset, *h,* are labeled.

A specific contact interaction process is shown in Figure 5. As an example, in Figure 4b, the microparticles were randomly distributed after drying. A series of manipulation steps were needed to organize them into ordered patterns. The manipulation process is briefly described below:

Step 1: Identify microparticle center.

Step 2: Move the probe tip to the initial position where the vertical distance on the center is 0.5 μm and the horizontal position is 1 μm, and then push the particle forward; when the microparticle slides away from the tip, the single-step push ends. When the microparticle is far away from the target position, the tip is put as close as possible to the microparticle center, thereby achieving maximum single-step distance and reducing the operations cycles. The push direction is always parallel to $\vec{AB}$.

Step 3: Move the tip backward and downward to locate the second pushing position. The tip must move back to avoid contact with the particle in the process of the downward movement. The set of pushing steps must be considered within the upper limit of error. When the microparticle gets close to its target, the offset, *h,* should be adjusted to tune the push direction, ensuring precise microparticle release. 

Step 4: Check if the particle has reached the target. Otherwise, go to Step 3. 

**Figure 6:**
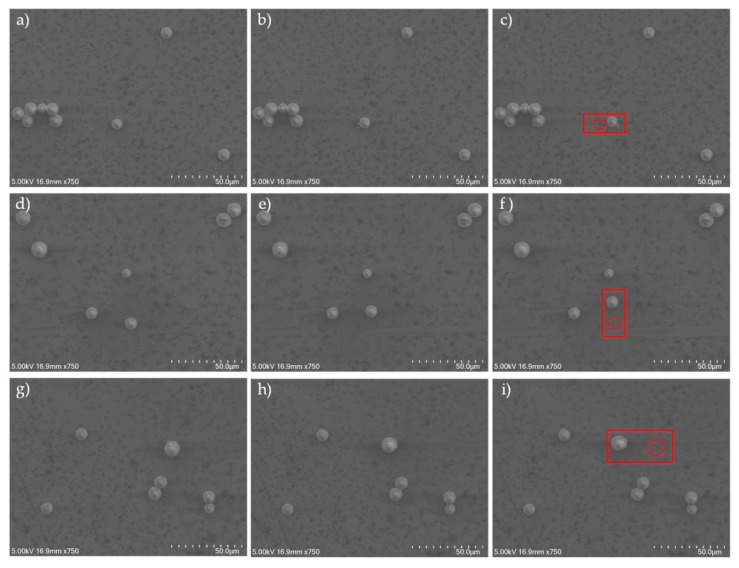
Pushing experiments using virtual nanohand strategy. The microparticle was pushed multiple steps from the beginning to its target. (**a**,**d**,**g**) Initial position. (**b**,**e**,**h**) Middle process. (**c**,**f**,**i**) Resultant position.

Three experimental pushes of microparticles, obtained with the proposed push strategy, are shown in Figure 6. The *y* displacements of the three results were 1.67 μm, 1.33 μm, and 3.93 μm. The *x* displacements of the three results were 8.83 μm, 15.33 μm, and 26 μm, respectively. The results show that the strategy of nano-hand is efficient and can obtain stable manipulation, especially for manipulation of microparticles over long distances. The current error could be reduced by next pushes.

**Figure 7:**
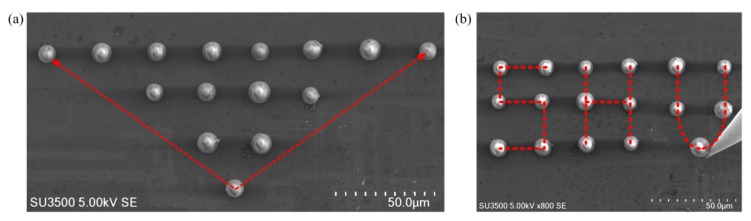
Well organized patterns by the proposed strategy. (**a**) A triangular microparticle matrix. (**b**) A ‘SHU’ microparticle matrix.

Figure 7 shows two organized patterns. During the whole process, no particle loss happened, and all the experimental results were finished in a very smooth way, which in turn proved the efficiency and effectiveness of the proposed strategy. For those two cases, average manipulation time was about 1 min per microparticle. The pushing speeds were kept at 2 μm/s, and manipulation time was variable depending on the distances between the initial particle locations and their destinations.

## 4. Discussion

Since the microparticles do not need to be attached to the tip like previous publications [34], the e-beam irradiation time is limited and the microparticles are well protected. At the same time, the proposed two-probe strategy can better protect the substrate and microparticles by reducing the pressure between the microparticles and the substrate. As a result, the original properties of the microparticles can be well maintained throughout the experiment because the microparticles are well protected.

### 4.1. h vs. Microparticle Single-Step Trajectory

**Figure 8:**
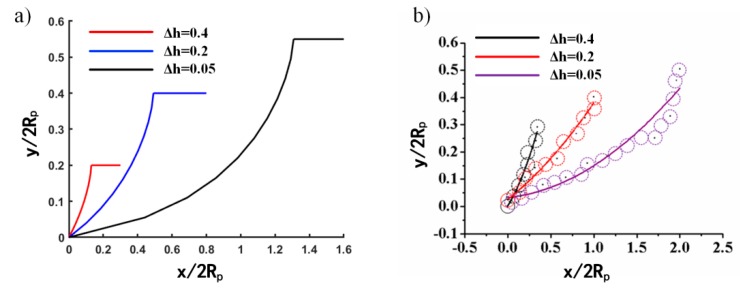
Theoretical (**a**) and experimental (**b**) microparticle trajectory with different offset, *h*.

The long-distance is finished by multiple single-step pushes. The tip poses are fixed, as the contact angle, *θ*, is fixed, and the microparticle trajectory will be dependent on *h*, according to Equation (4). As the microparticle sizes are variable, an initial relative offset, $\Delta h=h/R_{p}$ is defined for normalization. Trajectories of the microparticle center can be drawn by Matlab based on Equation 4, shown in Figure 8a.

In this experiment, the selected microparticles have a radius of 5 μm and a probe tip radius of 1 μm, so $\Delta Y_{max}=6\;$μm. The cut line in Figure 8a represents the separation position of the tip from the microparticle. 

To verify the theoretical microparticle trajectory, offsets, *h,* were set to 0.5 μm, 2 μm, and 4 μm, respectively. Experimental results show that the actual motion trajectory of the microparticles is in good agreement with theoretical predictions, which indicates that the model can predict the motion trajectory of the microparticles well, as shown in Figure 8. It can be seen from the actual trajectory that, the smaller offset h is, the larger displacement *x* is, consistent with the theory. Experiments have shown that when the microparticle center locates on the push path, the single-step displacement of the microparticle in the *x* direction will be 2–3 microparticle diameters.

### 4.2. Virtual Nano-Hand Strategy

It is known from the above single-step movement analysis that in most cases, the microparticle would deviate from the push path. By virtual nano-hand strategy, the motion error will be controlled in allowed limit, as shown in Figure 6. As no precise location is needed, as long as the displacement error is within control, the microparticle would be pushed on the same way.

**Figure 9:**
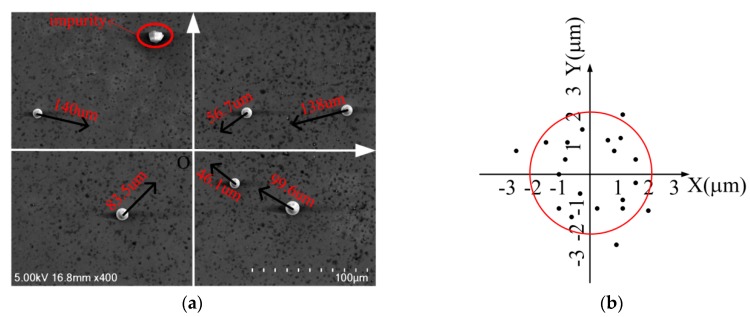
(**a**) The microparticles are to move to location *O*. (**b**) Final locations of microparticles around the target.

Since the virtual nano-hand strategy divides the long-distance path into multiple short paths, lengths of the whole path do not affect final release accuracy ideally. The final push step before settlement will determine the release precision of microparticles. Twenty sets of experiments were designed and implemented to prove release precision of the proposed strategies. The microparticles were pushed in the range of 100 μm in limited time respectively, and the final release position of each experimental microparticle was recorded, as shown in Figure 9, where final microparticle release positions and their target positions were shown.

Experimental results show that the probability of microparticle falling within 2 μm from the target is 80%, and the largest offset in the *x* direction is 2.51 μm, and the largest offset in the *y* direction is 2.69 μm. A total of 100% of the microparticles fell within 3 μm of the target. The release resolution could be improved by increasing push numbers, which would, however, increase operation time and cost. Though the yield has yet to improve, the experimental results proved the reliability and repeatability of the direct push protocol. 

### 4.3. Influencing Factors

In general, e-beam irradiation increases the adhesion of the microparticle during its movement. Accordingly, it was found that as the operation time increased, the probability of microparticles attaching to the tip increased. However, in cases of limited operation time, by reducing the SEM acceleration voltage and adjusting the interaction angle *θ*, the adsorption phenomenon could be minimized, and long-distance movement would be achievable. As the interaction between the microparticle and tip was reduced as much as possible, the tip wear was also reduced, and its life span was prolonged.

Pushing speed has yet to be optimized. In the nanomanipulator, the probe movement speed changes with SEM magnification, that is, the pushing speed is high as the magnification is small, and it is low as the magnification is high. The purpose is to allow the observer to observe the probe movement clearly. Increasing the pushing speed can speed up the manipulation; however, higher speed and clear observation require lower the magnification, which will increase the probe location error, and slow down manipulation. In addition, the microparticles may ‘fly’ away if the push speed is too high.

It was found in the experiment, that as operation duration and SEM acceleration voltage increases, the tip and microparticles occasionally appear mutually exclusive, which greatly affects the accuracy and efficiency of microparticle operation. How to avoid the charging of the microparticles in the vacuum chamber is also one of the subsequent research topics. 

## 5. Conclusions

Micro/nano-precise operation is still one of the most important issues in building micro/nano-blocks. This paper presents a new protocol for tip-based manipulation in SEM, which is based on the iterative use of successive directional push with two opposite probes. 

Aiming at position uncertainties during operation, the single-step trajectory of the microparticle is theoretically derived and verified by experimental results. The uncertainty of the microparticle is also overcome by a virtual nano-hand strategy. Tip parameters were adjusted in real-time according to the trajectory of the microparticles, and mimicked the effect of a more powerful and deterministic ‘hand’. The position error was always in control. To protect the substrate and improve efficiency, a double-tip operation strategy was also implemented. The advantage of these strategies was that the e-beam irradiation time was much reduced and the substrate and microparticles were better protected. Experimental results demonstrated that the proposed strategy is effective for tip-based nanomanipulation. Future work will extend the nanomanipulation to automated fabrication of designed patterns by adding automated push path planning.

## Supplementary Materials

The following are available online at https://www.mdpi.com/2072-666X/10/10/670/s1, Video S1: Two-probe strategy (playing speed × 4), Video S2: Virtual nano-hand strategy (playing speed × 8).
